# Routes to High-Performing Ruthenium–Iodide Catalysts for Olefin Metathesis: Ligand Lability Is Key to Efficient Halide Exchange

**DOI:** 10.1021/acs.organomet.1c00253

**Published:** 2021-06-16

**Authors:** Christian O. Blanco, Daniel L. Nascimento, Deryn E. Fogg

**Affiliations:** † Center for Catalysis Research & Innovation and Department of Chemistry and Biomolecular Sciences, University of Ottawa, Ottawa, ON, Canada K1N 6N5; ‡ Department of Chemistry, University of Bergen, Allégaten 41, N-5007 Bergen, Norway

## Abstract

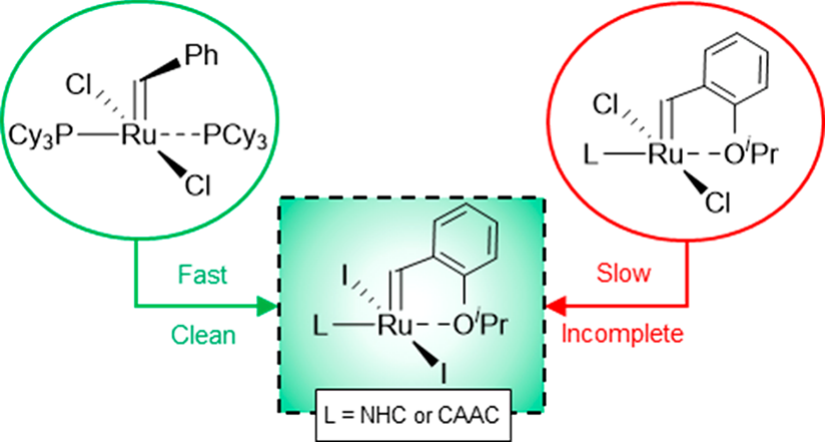

Clean, high-yielding routes are described to ruthenium–diiodide catalysts that were recently shown to enable high productivity in olefin metathesis. For the second-generation Grubbs and Hoveyda catalysts (**GII**: RuCl_2_(H_2_IMes)(PCy_3_)(=CHPh); **HII**: RuCl_2_(H_2_IMes)(=CHAr), Ar = C_6_H_4_-2-O*^i^*Pr), slow salt metathesis is shown to arise from the low lability of the ancillary PCy_3_ or ether ligands, which retards access to the four-coordinate intermediate required for efficient halide exchange. To exploit the lability of the first-generation catalysts, the diiodide complex RuI_2_(PCy_3_)(=CHAr) **HI-I**_**2**_ was prepared by treating “Grubbs I” (RuCl_2_(PCy_3_)_2_(=CHPh), **GI**) with NaI, H_2_C=CHAr (**1a**), and a phosphine-scavenging Merrifield iodide (**MF-I**) resin. Subsequent installation of H_2_IMes or cyclic (alkyl)(amino)carbene (CAAC) ligands afforded the second-generation iodide catalysts in good to excellent yields. Given the incompatibility of the nitro group with a free carbene, the iodo-Grela catalyst RuI_2_(H_2_IMes)(=CHAr′) (**nG-I**_**2**_: Ar′ = C_6_H_3_-2-O*^i^*Pr-4-NO_2_) was instead accessed by sequential salt metathesis of **GI** with NaI, installation of H_2_IMes, and finally cross-metathesis with the nitrostyrenyl ether H_2_C=CHAr′ (**1b**), with **MF-I** as the phosphine scavenger. The bulky iodide ligands improve the selectivity for macrocyclization in ring-closing metathesis.

Olefin metathesis is an exceptionally versatile methodology for the catalytic assembly of carbon–carbon bonds. It is now seeing attention in challenging contexts ranging from pharmaceutical manufacturing^1^ to chemical biology^2^ and materials science.^3^ While chlororuthenium catalysts (Chart 1) dominate these applications, iodide analogues offer important advantages.^4−6^ Long-overlooked because of their slower metathesis reactions,^7−9^ iodide catalysts such as **nG-I**_**2**_ have recently been shown to offer improved productivities in the synthesis of macrocycles via ring-closing metathesis (mRCM,^4,6^ a metathesis manifold of keen interest for the production of antiviral therapeutics),^10^ and increased selectivity for metathesis of terminal versus internal olefins.^5^ Their tolerance for ethylene^11,12^ (the coproduct in metathesis of terminal olefins) is also striking: it is due in part to relatively slow bimolecular decomposition.^6,12^ Indeed, their ethylene-tolerance is second only to that of cyclic (alkyl)(amino) carbene (CAAC) derivatives, examples of which appear in Chart 1.^13,14^ Heightened stability toward water^6^ adds further potential, most prominently for opportunities in chemical biology.

**Chart 1:**
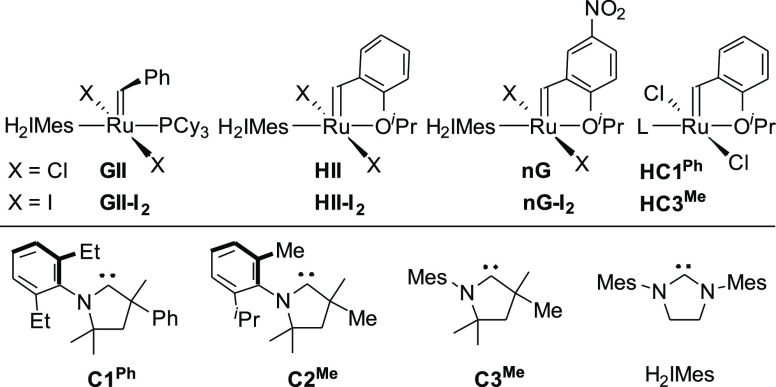
Olefin Metathesis Catalysts and NHC or CAACa Ligands Discussed

a In the CAAC identification system used, ligands are grouped into families (**C1**, **C2**, etc.) with a common NAr moiety. A superscript specifies the R substituent on the quaternary CMeR site α to the carbene carbon.

These advantages underscore the desirability of clean, general routes to the iodide catalysts. Inefficient halide exchange is reported even for some of the most successful published methods,^4,7,8^ in which second-generation catalysts were subjected to salt metathesis with KI in methanol (Table 1, entries 1−3). The limited solubility of the ruthenium reagents in methanol is one challenge,^17^ but less satisfactory results are reported in organic media (or on use of [*^n^*Bu_4_N]I instead of an alkali metal iodide).^8^ Slugovc has commented that equilibrium exchange in methanol results in incomplete reaction even after prolonged reaction and multiple workup stages.^7^ The presence of residual chloride catalyst is undesirable from the perspective of batch-to-batch reproducibility, robustness, and selectivity. In an alternative route, the Grubbs group described isolation of clean **GII-I_2_** following reaction of **GII** with NaI in THF^9^ (albeit in 75% isolated yield; whether mixed-halide species are present in the crude product was not discussed).

Here we demonstrate that a major obstacle to the transformation of Ru–carbene catalysts into their iodide analogues is the low lability^15^ of the neutral ancillary ligands that stabilize these complexes. Building on the higher lability of the PCy_3_-stabilized first-generation Grubbs and Hoveyda catalysts (**GI-I**_**2**_ and **HI-I**_**2**_, respectively),^16^ we report facile access to their iodide derivatives. The latter offer convenient platforms for the production of fully iodated, phosphine-free catalysts bearing N-heterocyclic carbene (NHC) or CAAC ligands. Finally, we describe advances in mRCM with CAAC–iodide catalysts.

From a prior mechanistic study with sodium methoxide,^17c^ we suspected that the lability of the *neutral* ancillary ligand in the Ru precursors might be the key to efficient halide exchange. To confirm this point, we examined reactions of NaI with a series of Grubbs-class catalysts, for which the PCy_3_ lability spans 5 orders of magnitude.^15^ In situ yields of the diiodide products at 1 h declined in the order **GI** > **GII** > **GII′** ≫ **GIIm** (Table 1, entries 5–8). These yields correspond to the established^15^ trend in rates of PCy_3_ dissociation, consistent with our proposal that salt metathesis is mediated by four-coordinate RuCl_2_(L)(=CHPh).^17c^ Of note, the corresponding experiment with the first-generation complex **HI** effected complete halide exchange, versus 6% for its H_2_IMes analogue **HII** (entries 9 and 10). These findings confirm that catalyst lability is critical to efficient halide exchange, and point toward the potential of the more labile first-generation catalysts as entry points to the target complexes.

**Table 1:** Salt Metathesis of Ru–Dichloride Complexes^a^

					distribution (%)			
entry	parent	solvent	reagent (equiv)	time (h)	Cl_2_	Cl/I	I_2_	ref
1	**HII**	MeOH	KI (30)	3	4	12	84	(8)
2	**HII**	MeOH	KI (25–30)	3–4 (4×)^b^	0	4	76	(7)
3	**nG**	MeOH	KI (30)	48 (2×)^b^	0	1	93	(4)
4	**GII**	THF	NaI (20)	8	NR^c^	NR	75	(9)
5	**GI**	THF	NaI (20)	1	0	0	100	TW^f^
6	**GII**	THF	NaI (20)	1	43	0	54	TW
7	**GII′**^d^	THF	NaI (20)	1	89	1	10	TW
8	**GIIm**^e^	THF	NaI (20)	1	100	0	0	TW
9	**HI**	THF	NaI (20)	1	0	0	100	TW
10	**HII**	THF	NaI (20)	1	94	6	0	TW

a All the reactions were performed at ambient temperature.

b Each cycle required removal of the solvent, isolation of the Ru species, washing, and resuspension in MeOH.

c NR = not reported.

d **GII′** = RuCl_2_(IMes)(PCy_3_)(=CHPh).

e **GIIm** = RuCl_2_(H_2_IMes)(PCy_3_)(=CH_2_).

f TW = this work.

A complementary line of inquiry was prompted by Morris’ successful use of a Merrifield iodide resin (**MF-I**) to synthesize the iodo catalyst RuHI(BINAP)(PPh_3_) from its chloride precursor.^18^ The reported behavior contrasts with our observation of selective PCy_3_ sequestration when **MF-I** was used to aid in the synthesis of second-generation olefin metathesis catalysts. That is, we observed phosphine scavenging with no competing iodation.^19^ The difference in reactivity of these aryl- and alkylphosphine complexes reinforced the importance of ligand lability for efficient halide exchange. It also raised the possibility of using **MF-I** to effect both PCy_3_ scavenging and halide exchange in first-generation systems.

Accordingly, we treated **GI** with 2-isopropoxystyrene (**1a**) in the presence of **MF-I** (Scheme 1a). To swell the resin,^20^ as required for rapid S_N_2 reaction with PCy_3_, we employed THF as the solvent. **GI** was completely consumed after 3 h at 50 °C, as judged by NMR analysis: the null ^31^P NMR spectrum confirmed successful sequestration of PCy_3_. However, despite a formal 6-fold excess of the resin −CH_2_I repeat unit, **HI-I**_**2**_ was formed in only 18% yield. Still present was 20% residual **HI** and 62% of the monoiodo species **HI-I**, a ratio that was unaffected by further reaction. We conclude that **MF-I** alone is inefficient in inducing complete halide exchange in the present system.

**Scheme 1:**
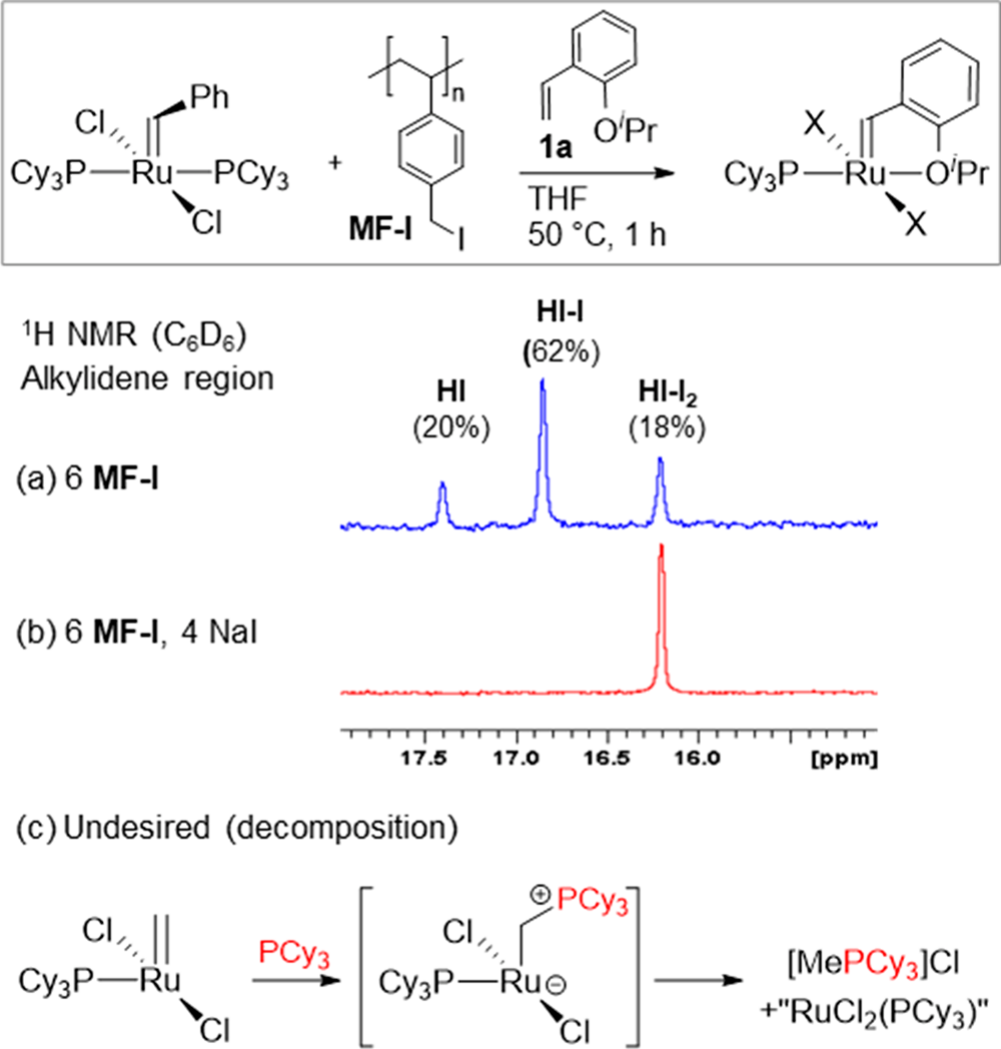
Synthesis of **HI-I**_**2**_ by Cross-Metathesis with **1a**: (a, b) Progress in the Presence of (a) **MF-I** Only or (b) **MF-I** and NaI; (c) Decomposition Reaction (Suppressed by Excess **MF-I**)

Conversion to **HI-I**_**2**_ could be completed by adding NaI (4 equiv) and stirring for 2 h at room temperature (RT). Workup involved evaporation of the solvent, redissolution in benzene, and filtration through Celite to remove residual salts and resin. Reprecipitation from benzene/hexanes afforded spectroscopically clean **HI-I**_**2**_ in 86% yield. Alternatively, **GI** could be treated simultaneously with styrenyl ether **1a**, **MF-I**, and NaI at 50 °C to effect complete exchange of the chloride and PCy_3_ ligands, as well as phosphine scavenging (Scheme 1b). Under these conditions, **HI-I**_**2**_ was observed within 3 h. Workup as before afforded **HI-I**_**2**_ in 82% yield, but slightly lower purity. In comparison, the Hoveyda group reported a 67% isolated yield of **HI** upon cross-metathesis of **GI** with **1a** in CH_2_Cl_2_, with chromatographic workup.^16^ The 15% improvement in yield in the present work is due in part to efficient interception of PCy_3_ by the resin, which prevents nucleophilic attack on the methylidene intermediate generated during catalysis (Scheme 1c).^21^ Importantly, no signal for [MePCy_3_]Cl was evident in the ^31^P{^1^H} NMR spectrum of the crude product (ca. 34 ppm, C_6_D_6_).^22^

With **HI-I**_**2**_ in hand, we explored its potential as a platform for the synthesis of second-generation diiodide catalysts by ligand exchange with an NHC or CAAC ligand. Three exemplary reactions were explored. **HII-I**_**2**_ (Scheme 2a) was generated by stirring **HI-I**_**2**_ with free H_2_IMes in THF at RT, and adding **MF-I** once coordination of the nucleophilic carbene was complete (1 h). No observable ^31^P NMR signals remained after 45 min. **HII-I**_**2**_ was isolated in 93% yield by filtration through Celite and evaporation of the solvent, with no need for chromatography or extraction.^23,24^ The cleanliness of this ligand-exchange reaction relative to olefin metathesis routes to second-generation catalysts is due to (1) the fact that no vulnerable methylidene or metallacyclobutane intermediates are generated and (2) the use of isolated free H_2_IMes.^25^

**Scheme 2:**
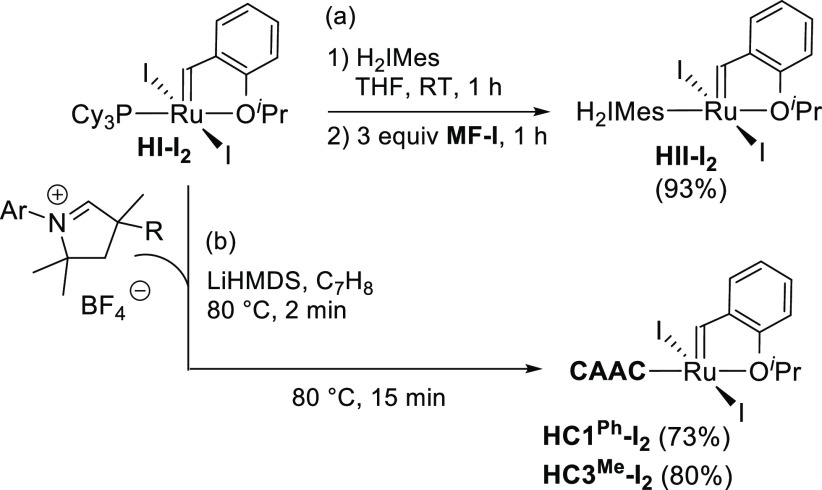
Ligand-Exchange Routes to Second-Generation Ru–Iodide Complexes: (a) **HII-I**_**2**_ Catalysts; (b) CAAC Catalysts

Within the corresponding CAAC catalysts, we examined the Hoveyda-class complexes of **C1**^**Ph**^ and **C3**^**Me**^, which offer extremes of steric bulk. Their synthesis is hampered by the instability of the free CAAC proligands, which must be generated in situ.^26^ Following the Skowerski method,^27^ we heated the CAAC·BF_4_ salts with LiHMDS in toluene at 80 °C for 2 min and then added **HI-I**_**2**_ and stirred for 15 min (Scheme 2b). The **MF-I** resin was not employed in workup since further purification would be required in any case, to remove salts and other byproducts with no affinity for the resin. Instead, silica-gel chromatography (1:2 CH_2_Cl_2_/hexanes) was conducted to remove all of the byproducts simultaneously. The target iodo catalysts were isolated in good yields (73% for **HC1**^**Ph**^**-I**_**2**_; 80% for **HC3**^**Me**^**-I**_**2**_).

A modified approach is required to access the corresponding nitro-Grela complex **nG-I**_**2**_ because the nucleophilic free carbene is incompatible with the −NO_2_ and −CH_2_I functionalities. While the Grela-class analogue of **HI-I**_**2**_ was readily accessible by the method of Scheme 1b (i.e., via metathesis of **GI** with H_2_C=CHAr′ (**1b**) (Ar′ = C_6_H_3_-2-O^i^Pr-4-NO_2_) in the presence of NaI and **MF-I**), addition of H_2_IMes resulted in immediate decomposition. Clearly, the NHC ligand must be installed prior to nitrostyrenyl ether **1b**. The three-step sequence shown in Scheme 3 accommodates this requirement, as well as the lower rate of halide exchange relative to PCy_3_ exchange (which introduces the potential for competing reaction of free H_2_IMes with the resin). Accordingly, **GI** was stirred with 20 equiv of NaI in THF (step 1) for 2 h, and once complete halide exchange was verified, free H_2_IMes was added (step 2). After 1 h, ligand exchange was complete, and nitrostyrenyl ether **1b** and **MF-I** were added (step 3) to effect the final cross-metathesis step and sequester free PCy_3_.^28^ Workup as above yielded **nG-I**_**2**_ in 77% net yield over the three reactions, namely, salt metathesis, installation of the NHC, and cross-metathesis.

**Scheme 3:**
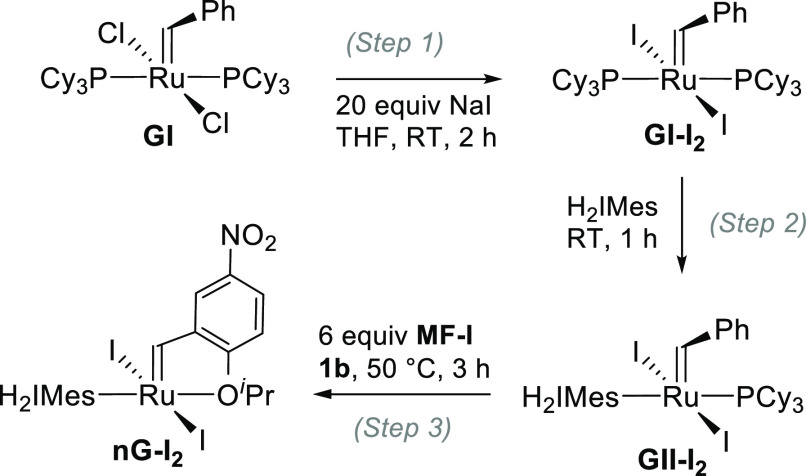
One-Pot Synthesis of **nG-I**_**2**_ from **GI**

The new catalysts hold intriguing potential in mRCM in view of evidence that bulky ligands accelerate cyclization of conformationally flexible dienes (e.g., musk precursor **2**; Table 2).^29^ Also important is the slower bimolecular decomposition of RuI_2_(L)(=CH_2_).^12^ Catalyst lifetime is critical for such mRCM reactions because cyclization typically proceeds via a concentration-dependent ring–chain equilibrium, in which oligomerization is kinetically preferred and the oligomers liberate the desired products via backbiting.^30^ For dienes with little conformational bias toward cyclization (in diene **2**, the ester group provides the sole such bias),^31^ dilutions of ≤5 mM can be required to shift the equilibrium in favor of cyclic products. Importantly, ligand bulk appears to accelerate the slow backbiting step,^29^ in addition to retarding decomposition.

**Table 2:**
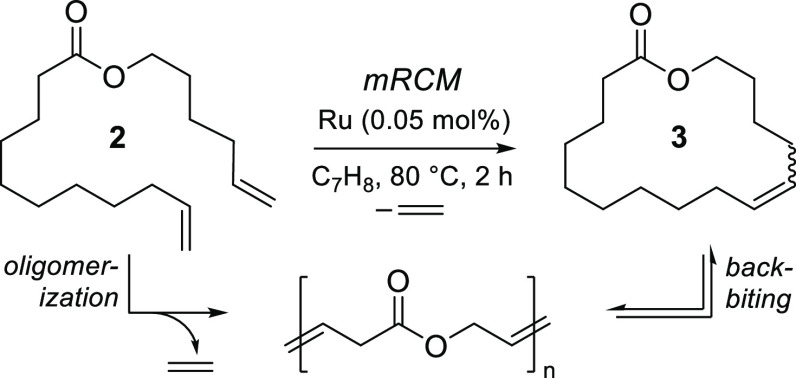
mRCM Performance of Iodide versus Chloride Catalysts

catalyst	initial [**2**] (mM)	% conv.	% mRCM	% oligomer
**HC1**^**Ph**^	5	100	87	13
**HC1**^**Ph**^**-I**_**2**_	5	100	100	0
**HC3**^**Me**^**-I**_**2**_	5	97	91	6
**HC1**^**Ph**^	20	100	68	32
**HC1**^**Ph**^**-I**_**2**_	20	100	76	24
**HC3**^**Me**^**-I**_**2**_	20	100	86	14
**nG**	20	100	74	26
**nG-I**_**2**_	20	97	85	12

The diiodide catalysts **HC1**^**Ph**^**-I**_**2**_ and **HC3**^**Me**^**-I**_**2**_ were thus screened alongside chloride catalyst **HC1**^**Ph**^ in mRCM of **2** at 80 °C (Table 2). At 0.05 mol % Ru, **HC1**^**Ph**^**-I**_**2**_ effected quantitative formation of macrocycle **3** within 2 h, vs 87% mRCM and 13% oligomers for **HC1**^**Ph**^. Intermediate performance was seen for **HC3**^**Me**^**-I**_**2**_, with its smaller CAAC ligand. To test whether the iodide ligands confer a kinetic bias toward cyclization (i.e., selectivity for direct mRCM), these reactions were repeated at 20 mM **2**. Oligomers were seen in all cases (though less so for **HC1**^**Ph**^**-I**_**2**_ and **HC3**^**Me**^**-I**_**2**_ than **HC1**^**Ph**^), indicating that the kinetic preference for intermolecular reaction is retained. Interestingly, **HC3**^**Me**^**-I**_**2**_ emerged as the most productive at this concentration, affording **3** in 86% yield. The NHC catalyst **nG-I**_**2**_ shows similarly improved mRCM selectivity relative to its chloride analogue **nG**. Incorporation of iodide ligands may thus improve the selectivity for mRCM even where high dilutions are impractical.^32^

The foregoing describes clean routes to phosphine-free ruthenium–diiodide metathesis catalysts, via the synthesis and use of first-generation catalysts as labile platforms for subsequent modification. **HI-I**_**2**_ is conveniently prepared by salt metathesis with NaI in THF, using the Merrifield iodide resin **MF-I** to scavenge the PCy_3_ coproduct. Second-generation Hoveyda-class catalysts can then be obtained by ligand exchange with H_2_IMes or CAAC ligands. For nitro-Grela derivatives, **GI-I**_**2**_ offers a suitable entry point, but installation of the carbene via ligand exchange must then precede installation of the nitrobenzylidene functionality. The iodide catalysts were shown to improve the selectivity for cyclic products in macrocyclization, one of the key current applications of olefin metathesis. These findings are expected to further advance the development of highly robust, productive catalysts for olefin metathesis.

## Supporting Information Available

Experimental details and NMR spectra (PDF)
