# Supplementary material for: Routes to High-Performing Ruthenium–Iodide Catalysts for Olefin Metathesis: Ligand Lability Is Key to Efficient Halide Exchange
Source: Organometallics. 2021 Jun 16;40(12):1811–6. doi: 10.1021/acs.organomet.1c00253 (PMC8289337; doi:10.1021/acs.organomet.1c00253)
Supplement: Supplementary file 1 — om1c00253_si_001.pdf [file om1c00253_si_001.pdf]

Supporting Information

Routes to High-Performing Ruthenium-Iodide Catalysts for Olefin Metathesis: Ligand Lability Is Key to Efficient Halide Exchange

Christian O. Blanco,[a] Daniel L. Nascimento,[a] and Deryn E. Fogg*[a,b]

[a]Center for Catalysis Research & Innovation, and Department of Chemistry and Biomolecular Sciences, University of Ottawa, ON, Canada, K1N 6N5. [b]Department of Chemistry, University of Bergen, Allégaten 41, N-5007 Bergen, Norway

*Corresponding author: dfogg@uottawa.ca, dfo025@uib.no

Table of Contents

S1. Experimental.......... S2
S2. NMR Spectra.......... S7
S3. References.......... S16

S1. Experimental.

General Procedures: All reactions were carried out under N_2 in a glovebox. HPLC-grade CH_2Cl_2, C_6H_6, hexanes, and THF were dried and degassed using a Glass Contour solvent purification system, and stored under N_2 over 4 Å molecular sieves for at least 24 h prior to use. Toluene was distilled over Na and stored as above. Liquid reagents (**1a**, **1b**, **2**), dodecane (GC internal standard; Sigma, anhydrous, 99%) and C_6D_6 (Cambridge Isotopes), were freeze-pump-thaw degassed (4×), and stored under N_2 in the glovebox; C_6D_6 was stored over sieves as above. NaI (99.5%), dimethyl terephthalate (DMT, IS) and Merrifield resin (**MF-Cl**) (4.5 mmol•g^{-1} chloride loading; 1% cross-linked with divinylbenzene; 200–400 mesh) were purchased from Sigma-Aldrich and used as received. Ruthenium precursor **GI**,[1] CAAC salts (**C1^{Ph}**•BF_4, **C3^{Me}**•BF_4),[2] 2-isopropoxystyrene **1a**,[3] 2-isopropoxy-4-nitrostyrene **1b**,[3] diene **2**,[4] lactone **3**,[4] the Merrifield iodide resin (**MF-I**)[5] and H_2IMes[6] were prepared by literature methods. All but **GI** and the CAAC salts were stored in the glovebox freezer at –35 ° C.

NMR spectra were recorded on Bruker Avance 300 MHz, Bruker Avance III 500 MHz or Bruker Avance III 600 MHz spectrometers at 25 ±2 °C. Chemical shifts are given in ppm and referenced to the residual proton of the deuterated solvent (^{1}H NMR) or 85% external H_3PO_4 (^{31}P NMR; 0 ppm). For new compounds, proof of identity is provided by ESI mass spectrometric analysis, collected on a Waters Acquity TQD mass spectrometer. Samples were dissolved in MeCN in inert atmosphere and injected via syringe pump (30 uL/min). Spectra were obtained with a capillary voltage of 3.5 kV, a cone voltage of 25 V, a source temperature of 80 °C, and desolvation gas temperature of 180 °C. Both desolvation gas flow and cone gas flow were 100 L/min. The presence of a single unique product was confirmed by chromatography and confirmed by NMR analysis. Fully-assigned ^{1}H NMR spectra with numbered ^{1}H nuclei, consistent with the proposed structures, appear in Section S2.

Reactions involving macrocyclization by ring-closing metathesis (mRCM) were analyzed using an Agilent 7890A gas chromatograph (GC) equipped with auto-sampler, flame ionization detector (FID) and Agilent HP-5 polysiloxane column (30 m length, 320 μm diameter). Helium (UHP grade) was used as the carrier gas to maintain column pressure at 11.5 psi. Calibration curves of peak areas versus concentration were established for diene **2** and cyclic product **3** in the relevant concentration regimes, with ca. 1:1 (w/w) sample versus dodecane as internal standard.

Note: In formulae shown below, Ar = C_6H_4-2-O^{i}Pr; Ar' = C_6H_4-2-O^{i}Pr-4-NO_2.

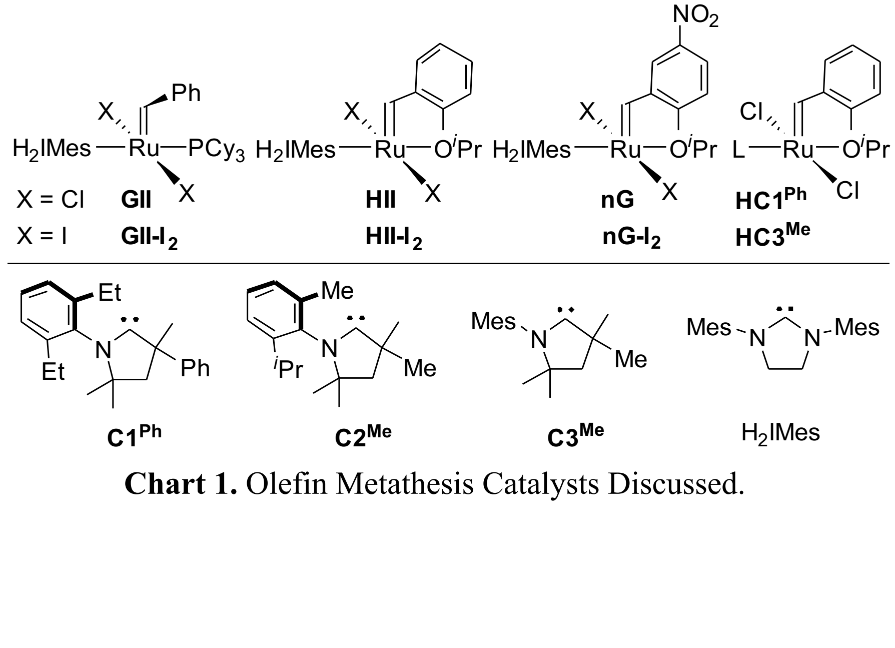

Chart 1. Olefin Metathesis Catalysts Discussed.

First-Generation Hoveyda Catalyst $RuI_2(PCy_3)_2(=CHAr)$ (HI-I_2; Ar = C_6H_4-2-O^iPr).

Synthesis from GI by Sequential Cross-Metathesis and Salt Metathesis (Optimal Route). To a purple solution of **GI** (250 mg, 0.300 mmol) in 10 mL THF was added 2-isopropoxystyrene **1a** (55 mg, 0.33 mmol, 1.1 equiv) and the **MF-I** resin (600 mg, 1.80 mmol, 6 equiv). The reaction was stirred in a sealed Schlenk flask at 50 °C (degassed oil bath, glovebox), with periodic NMR analysis. A colour change to green-brown occurred within 15 min. After 3 h, no observable **GI** remained (NMR). The resin was filtered off (Celite: this filtration step may be omitted for convenience), and product was washed through with THF (3 × 5 mL). Addition of solid NaI (910 mg, 6.00 mmol, 20 equiv) to the filtrate caused the colour to deepen to dark green over 15 min at RT. Stirring was continued for 2 h, when full conversion to **HI-I_2** was evident (NMR). The solvent was evaporated to dryness. The residue was taken up in benzene (5 mL) and the Na salts were filtered off, washing through with further benzene (4 × 5 mL). The combined filtrate was concentrated to a minimum volume, and cold hexanes was added to precipitate the product as a green solid, which was filtered off, washed with hexanes (3 × 1 mL), and dried under vacuum. Yield of clean **HI-I_2**: 202 mg, 0.258 mmol (86%).

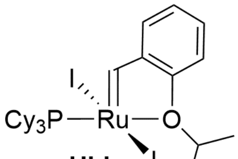

HI-I_2

^{1}H NMR (500 MHz, C_6D_6): 16.24 (d, $^3J_{HP}$ = 4.1 Hz, 1H, [Ru]=C*H*), 7.38 (dd, $^3J_{HH}$ = 7.6 Hz, $^4J_{HH}$ = 1.6 Hz, 1H, Ar C*H*), 7.23 (dd, $^3J_{HH}$ = 7.8 Hz, $^3J_{HH}$ = 1.2 Hz, 1H, Ar C*H*), 6.60 (m, 2H, Ar C*H*), 4.95 (sept, $^3J_{HH}$ = 12.4 Hz, 1H, C*H*Me$_2$), 2.63 (m, 3H, Cy C*H*), 2.26 (br, 6H, Cy), 1.98 (br, 6H, Cy), 1.83 (d, $^3J_{HH}$ = 7.4 Hz, 6H, CH(C*H*$_3$)$_2$), 1.74 (br, 6H, Cy), 1.58 (br, 2H, Cy), 1.23 (br, 10H, Cy). For fully-assigned ^{1}H NMR spectrum, see Figure S1.

^{13}C{^{1}H} NMR (125 MHz, C_6D_6): δ 274.4 ([Ru]=*C*; not observed: detected by ^{1}H-^{13}C HSQC), 153.1, 143.8, 129.7, 122.9, 121.9, 114.1, 76.4, 39.5, 39.3, 31.4, 27.8, 27.7, 26.3, 22.5. ^{31}P{^{1}H} NMR (200 MHz, C_6D_6): δ 72.2. ESI-MS (MeCN): Calcd for $C_{28}H_{45}IOPRu$ ([M–I]$^+$), *m/z* 657.2689. Found: *m/z* 657.1296.

Alternative Route to HI-I_2 via Simultaneous Cross-Metathesis and Salt Metathesis. To a mixture of **GI** (250 mg, 0.300 mmol), **MF-I** resin (660 mg, 1.80 mmol, 6 equiv) and NaI (180 mg, 1.2 mmol, 4 equiv) in 10 mL THF was added 2-isopropoxystyrene **1a** (55 mg, 0.33 mmol, 1.1 equiv). The suspension was stirred in a sealed Schlenk flask at 50 °C (degassed oil bath, glovebox). A colour change from purple to yellow-brown occurred within 15 min. At 1 h, full conversion to **HI-I_2** was evident. Workup as above gave **HI-I_2** in slightly lower yields and purity than above. Yield: 192 mg, 0.245 mmol (82%).

"Second-Generation" NHC and CAAC Iodide Derivatives

Synthesis of Known $RuI_2(H_2IMes)(=CHAr)$, HII-I_2 from HI-I_2 by Ligand Exchange. Solid **HI-I_2** (500 mg, 0.630 mmol) and white solid H_2IMes (214 mg, 0.700, 1.1 equiv) were dissolved in 10 mL THF. The green solution was stirred for 1 h, at which point formation of **HII-I_2** was complete (NMR). The **MF-I** resin (630 mg, 1.89 mmol, 3 equiv) was added, and stirring was continued for 1 h. The resin was then filtered off (Celite), product was washed through with THF (3 × 10 mL), the combined filtrate was evaporated, and the green solid was washed with hexanes (3 × 3 mL). Yield of clean **HII-I_2**: 480 mg, 0.593 mmol (93%). NMR data in C_6D_6 are in general agreement with

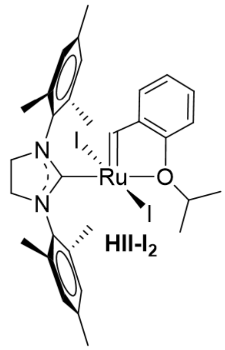

HII-I_2

values reported in $CDCl_3$ (*note*: in $CDCl_3$, the key Ru=C*H*Ar singlet appears at 15.66 ppm).[7]

^{1}H NMR (500 MHz, C_6D_6): δ 15.85 (s, 1H, Ru=C*H*Ar), 7.11 (m, Ar; integration hampered by overlap with C_6D_5H), 6.95 (s, 2H, Mes C*H*), 6.80 (s, 2H, Mes C*H*), 6.56 (m, 1H, Ar C*H*), 6.39 (d, $^3J_{HH}$ = 8.2 Hz, 1H, Ar C*H*), 4.66 (sept, $^3J_{HH}$ = 6.1 Hz, 1H, C*H*Me$_2$), 3.41 (s, 4H, NHC NCH_2), 2.80 (s, 6H, Mes CH_3), 2.57 (s, 6H, Mes CH_3), 2.20 (s, 6H, Mes CH_3), 1.45 (d, $^3J_{HH}$ = 5.8 Hz, 6H, ^{i}Pr CH_3). For fully-assigned ^{1}H NMR spectrum, see Figure S2.

Synthesis of RuI$_2$(C1^{Ph})(=CHAr), HC1^{Ph}-I$_2$. White solid **C1^{Ph}**•BF$_4$ (520 mg, 1.28, 2 equiv) and LiHMDS (214 mg, 1.28 mmol, 2.0 equiv) were suspended in 5 mL toluene and stirred at 80 °C for 2 min. Green **HI-I$_2$** (500 mg, 0.640 mmol) was added and the resulting brown-yellow suspension was stirred at 80 °C for 15 min. The solvent was evaporated under reduced pressure and the dark-yellow oil was purified by silica-gel chromatography in air (1:2 CH_2Cl_2:hexanes). A green band was isolated. After evaporating the solvent, the resulting green solid was stirred with hexanes (15 min). The suspension was cooled to –20 °C, filtered off, washed with cold hexanes (3 × 3 mL), and dried in vacuo. Yield of green **HC1^{Ph}-I$_2$**: 373 mg, 0.452 mmol (73%).

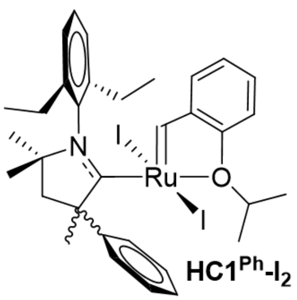

^{1}H NMR (500 MHz, C_6D_6): δ 15.49, (s, 1H, [Ru]=C*H*), 8.46 (d, $^3J_{HH}$ = 9.1 Hz, 2H, Ph *o*-C*H*), 7.56 (m, 2H, Ph *m*-C*H*), 7.34-7.25 (m, 2H, NAr + Ph *p*-C*H*), 7.21 (dd, $^3J_{HH}$ = 7.1 Hz, $^4J_{HH}$ = 2.0 Hz, 2H, NAr), 7.12 (dd, $^3J_{HH}$ = 9.1 Hz, $^4J_{HH}$ = 2.1 Hz, 1H, Ar C*H*), 6.95 (dd, $^3J_{HH}$ = 7.4 Hz, $^4J_{HH}$ = 1.7, 1H, Ar C*H*), 6.55 (m, 1H, Ar C*H*), 6.45 (d, $^3J_{HH}$ = 8.4 Hz, 1H, Ar C*H*), 4.72 (sept, $^3J_{HH}$ = 6.0 Hz, 1H, C*H*Me$_2$), 3.27 (m, 1H, C*H*HMe; diastereotopic), 3.02 (m, 1H,C*H*HMe; diastereotopic), 2.89 (d, $^3J_{HH}$ = 12.6 Hz, 1H, CAAC backbone C*H*H), 2.67 (m, 1H, C*H*HMe; diastereotopic), 2.45-2.52 (m, 4H, overlapping CH_3 + C*H*HMe; diastereotopic), 1.77 (d, $^3J_{HH}$ = 6.5 Hz, 3H, ^{i}Pr CH_3), 1.73 (d, $^3J_{HH}$ = 13.5 Hz, 1H, CAAC backbone C*H*H), 1.62 (d, $^3J_{HH}$ = 6.5 Hz, 3H, ^{i}Pr CH_3), 1.19 (t, $^3J_{HH}$ = 7.8 Hz, 3H, CH$_2$CH_3), 1.08 (s, 3H, CH_3), 1.00 (s, 3H, CH_3), 0.92 (t, $^3J_{HH}$ = 7.8 Hz, 3H, CH$_2$CH_3). For fully-assigned ^{1}H NMR spectrum, see Figure S3.

^{13}C{^{1}H} NMR (125 MHz, C_6D_6): δ 297.4 ([Ru]=*C*H), 267.9 (CAAC *C*:), 153.1, 147.8, 144.1, 144.1, 143.4, 139.2, 121.2, 113.9, 77.1, 75.0, 64.2, 45.2, 34.2, 31.2, 26.9, 26.4, 24.9, 23.0, 22.9(5), 15.7, 14.6.

ESI-MS (MeCN): Calcd for $C_{27}H_{37}INORu$ ([M–I]$^+$), *m/z* 696.1276. Found: *m/z* 696.2605.

Synthesis of RuI$_2$(C3^{Me})(=CHAr), HC3^{Me}-I$_2$. Prepared as for **HC3^{Me}-I$_2$**, using **HI-I$_2$** (250 mg, 0.320 mmol), **C3^{Me}**•BF$_4$ (212 mg, 0.640, 2 equiv), LiHMDS (107 mg, 0.640 mmol, 2.0 equiv), Yield of dark-green **HC3^{Me}-I$_2$**: 190 mg, 0.253 mmol (80%).

^{1}H NMR (500 MHz, C_6D_6): δ 15.30 (s, 1H, [Ru]=C*H*), 7.15 (s, Ar; integration hampered by overlap with C_6D_5H), 7.05 (dd, $^3J_{HH}$ = 7.1 Hz, $^4J_{HH}$ = 1.5 Hz, 1H, Ar C*H*), 6.77 (s, 2H, Mes *m*-C*H*), 6.58 (m, 1H, Ar C*H*), 6.47 (d, $^3J_{HH}$ = 9.5 Hz, 1H, Ar C*H*), 4.83 (sept, $^3J_{HH}$ = 5.6 Hz, 1H, C*H*Me$_2$), 2.42 (s, 6H, CH_3), 2.29 (s, 6H, CH_3), 2.15 (s, 3H, Mes *p*-CH_3), 1.80 (d, 6H, $^3J_{HH}$ = 5.9 Hz, ^{i}Pr CH_3), 1.76 (s, 2H, CAAC backbone CH_2), 1.02 (s, 6H, Mes *o*-CH_3). For fully-assigned ^{1}H NMR spectrum, see Figure S4.

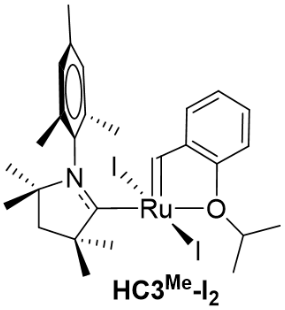

^{13}C{^{1}H} NMR (125 MHz, C_6D_6): 293.6 ([Ru]=*C*H), 270.4 (CAAC *C*:), 153.0, 144.6, 138.3, 137.4, 137.5, 130.4, 123.6, 121.2, 113.7, 78.3, 75.6, 54.7, 51.0, 32.6, 28.3, 22.9, 22.4, 20.4.

ESI-MS (MeCN): Calcd for $C_{33}H_{41}INORu$ ($[M–I]^+$), *m/z* 620.0963. Found: *m/z* 620.2942.

Grela Catalysts.

First-Generation Grela Catalyst $RuI_2(PCy_3)(=CHAr')$ (nGI-I_2; Ar' = C_6H_4-2-O^iPr-4-NO_2). Synthesis by Sequential Cross-Metathesis and Salt Metathesis. To a purple solution of **GI** (500 mg, 0.610 mmol) in 10 mL THF was added 2-isopropoxy-4-nitrostyrene **1b** (140 mg, 0.67 mmol, 1.1 equiv) and the **MF-I** resin (1.22 g, 3.66 mmol, 6 equiv). The resulting suspension was stirred in a sealed Schlenk flask at 50 °C (degassed oil bath, glovebox), with periodic NMR analysis. A colour change to yellow-brown occurred within 15 min. After 3 h, no NMR signals for **GI** were apparent. The resin was filtered off (Celite), and additional product was washed through with THF (3 × 5 mL). On adding solid NaI (1.83 mg, 12.2 mmol, 20 equiv), the stirred suspension turned dark green within 15 min at RT. After 2 h, full conversion to **nGI-I_2** was evident (NMR). The solvent was evaporated to dryness, and the residue was taken up in benzene (5 mL) and filtered to remove the Na salts. The product was washed through with benzene (3 × 5 mL). The combined filtrate was concentrated to a minimum volume, and hexanes was added to precipitate the green product, which was filtered off, washed with cold hexanes (3 × 1 mL), and dried under vacuum. Yield of **nGI-I_2**: 280 mg, 0.511 mmol (85%).

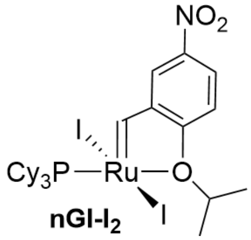

nGI-I_2

^1H NMR (300 MHz, C_6D_6): δ 15.81 (d, $^3J_{HP}$ = 3.7 Hz, 1H, [Ru]=C*H*), 8.19 (d, $^3J_{HH}$ = 2.2 Hz, 1H, Ar'), 8.01 (dd, $^3J_{HH}$ = 9.1 Hz, $^4J_{HH}$ = 2.1 Hz, 1H, Ar'), 6.20 (d, $^3J_{HH}$ = 9.5 Hz, 1H, Ar'), 4.76 (sept, $^3J_{HH}$ = 5.5 Hz, 1H, C*H*Me$_2$), 2.52 (m, 3H, Cy C*H*), 2.14 (br m, 6H, Cy CH_2), 1.99-1.45 (br, 20H, overlapping ^{i}Pr CH_3 + Cy CH_2), 1.20 (m, 10H, Cy CH_2). For fully-assigned ^1H NMR spectrum, see Figure S5.

$^{13}C\{^1H\}$ NMR (125 MHz, C_6D_6): δ 268.2 ([Ru]=*C*H, not observed; located by ^1H-^{13}C HSQC), 156.3, 142.9, 142.6, 124.1, 117.5, 113.4, 78.5, 39.5, 39.3, 31.3, 27.7, 27.6, 26.1, 22.4.

$^{31}P\{^1H\}$ NMR (200 MHz, C_6D_6): δ 73.8.

ESI-MS (MeCN): Calcd for $C_{28}H_{44}INO_3PRu$ ($[M–I]^+$), *m/z* 702.1147. Found: *m/z* 702.1952.

Second-Generation Catalyst nG-I_2.

Synthesis via Simultaneous Cross-Metathesis and Salt Metathesis. Solid purple **GI** (100 mg, 0.120 mmol) in 10 mL THF was stirred with NaI (359 mg, 2.40 mmol, 20 equiv) for 2 h. The dark-brown suspension was filtered (Celite) to remove the salts, washing through with THF (3 × 3 mL). Solid H_2IMes (41 mg, 0.13 mmol, 1.1 equiv) was then added and the dark-brown solution was stirred at RT for 1 h, at which point ligand exchange was complete (NMR). The **MF-I** resin (243 mg, 0.73, 6 equiv) was added, and the reaction was stirred for 1 h. The resin was then filtered off (Celite), and the product was washed through with THF (3 × 3 mL). To the filtrate was added 2-isopropoxy-4-nitrostyrene **1b** (27 mg, 0.13 mmol, 1.1 equiv) and fresh **MF-I** (243 mg, 0.73, 6 equiv). After stirring for 3 h at 50 °C, complete conversion was confirmed (NMR). The resin was filtered off as before; the filtrate was concentrated to a minimum volume of benzene, and hexanes was added to precipitate the product as a dark-brown microcrystalline solid. The latter was filtered off, washed with cold hexanes (3 × 1 mL), and

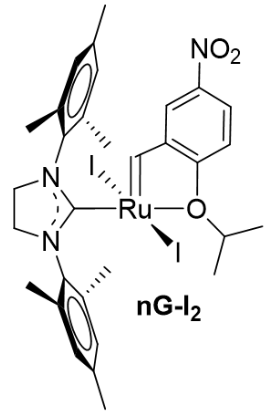

nG-I_2

dried under vacuum. Yield of **nG-I$_2$**: 79 mg, 0.092 mmol (77%). NMR data in C_6D_6 are in general agreement with the reported values in $CDCl_3$ (the key Ru=C*H*Ar signal in $CDCl_3$ appears at 15.51 ppm).[8]

^{1}H NMR (500 MHz, C_6D_6): δ 15.48 (s, 1H, Ru=C*H*Ar'), 7.87 (m, 2H, Ar'), 6.93 (s, 2H, Mes C*H*), 6.80 (s, 2H, Mes C*H*), 5.96 (d, 1H, $^3J_{HH}$ = 9.1 Hz, Ar'), 4.47 (sept, 1H, $^3J_{HH}$ = 6.0 Hz, C*H*Me$_2$), 3.37 (s, 4H, NHC NCH_2), 2.74 (s, 6H, Mes CH_3), 2.49 (s, 6H, Mes CH_3), 2.36 (s, 3H, Mes CH_3), 2.19 (s, 3H, Mes CH_3), 1.35 (d, 6H, $^3J_{HH}$ = 6.0 Hz, ^{i}Pr CH_3). For fully-assigned ^{1}H NMR spectrum, see Figure S6.

Examining Correlation Between Phosphine Lability and Salt Metathesis. See Table 1 in main text. In a representative procedure, **GI** (10 mg, 0.018 mmol) and DMT (internal standard; 5 mg, 0.025 mmol, 0.72 equiv) were dissolved in 1.50 mL THF and an aliquot was withdrawn, evaporated and redissolved in C_6D_6 to establish the initial ratio of **GI** to DMT (NMR). NaI (54 mg, 0.36 mmol, 20 equiv) was added. NMR analysis after stirring for 1 h: (a) **GI-I$_2$**: 100%. (b) **GII:** 43%, **GII-I$_2$**: 54%. (c) **GII'**: 89%, **GII'-I**: 1%, **GII'-I$_2$**: 10%. (d) **GIIm**: 100%. (e) **HI-I$_2$**: 100%. (f) **HII:** 94%, **HII-I**: 6%.

Representative Procedure for mRCM of Diene 2. A mixture of prolactone **2** (15 µL, 0.050 mmol) and dodecane (11 µL, 0.050 mmol, 1 equiv; internal standard for GC analysis) was dissolved in 2.45 mL C_7H_8; final concentration 5 mM **2**. A 150 µL aliquot was removed for GC-FID analysis to establish the starting ratio of **2** to dodecane. **HC1^{Ph}-I$_2$** (19 µL of a stock solution of 10 mg **HC1^{Ph}-I$_2$** in 10.0 mL C_6H_6; 0.025 µmol, 0.05 mol%) was added to the stirred solution, which was heated (glovebox, degassed oil bath) at 80±1 °C. Aliquots were removed periodically, quenched with KTp in THF (10 mg/mL; 10 equiv vs starting Ru) and analyzed by GC-FID. Yields of **3** are shown in Table 2 of the main text.

S2. NMR Spectra.

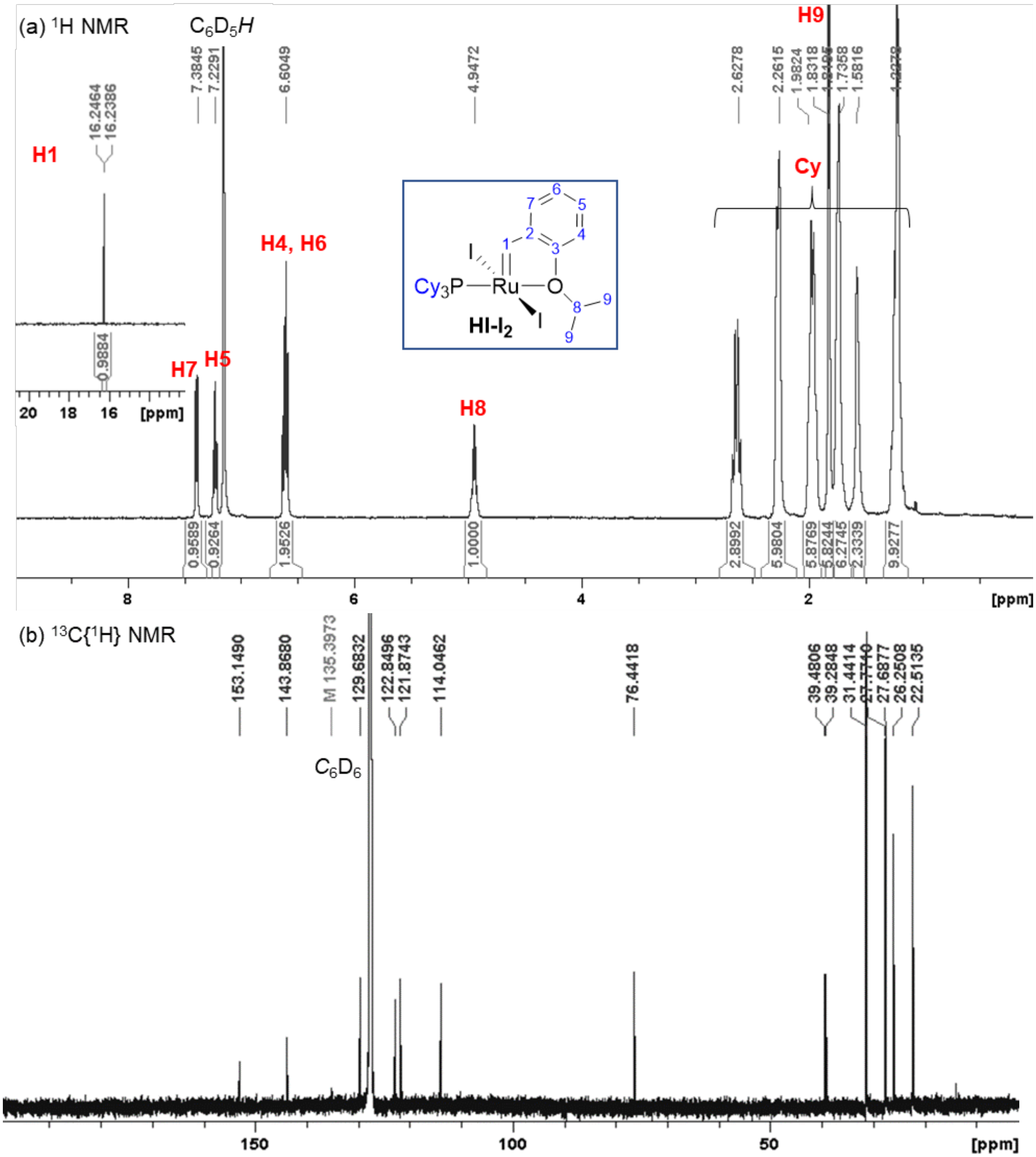

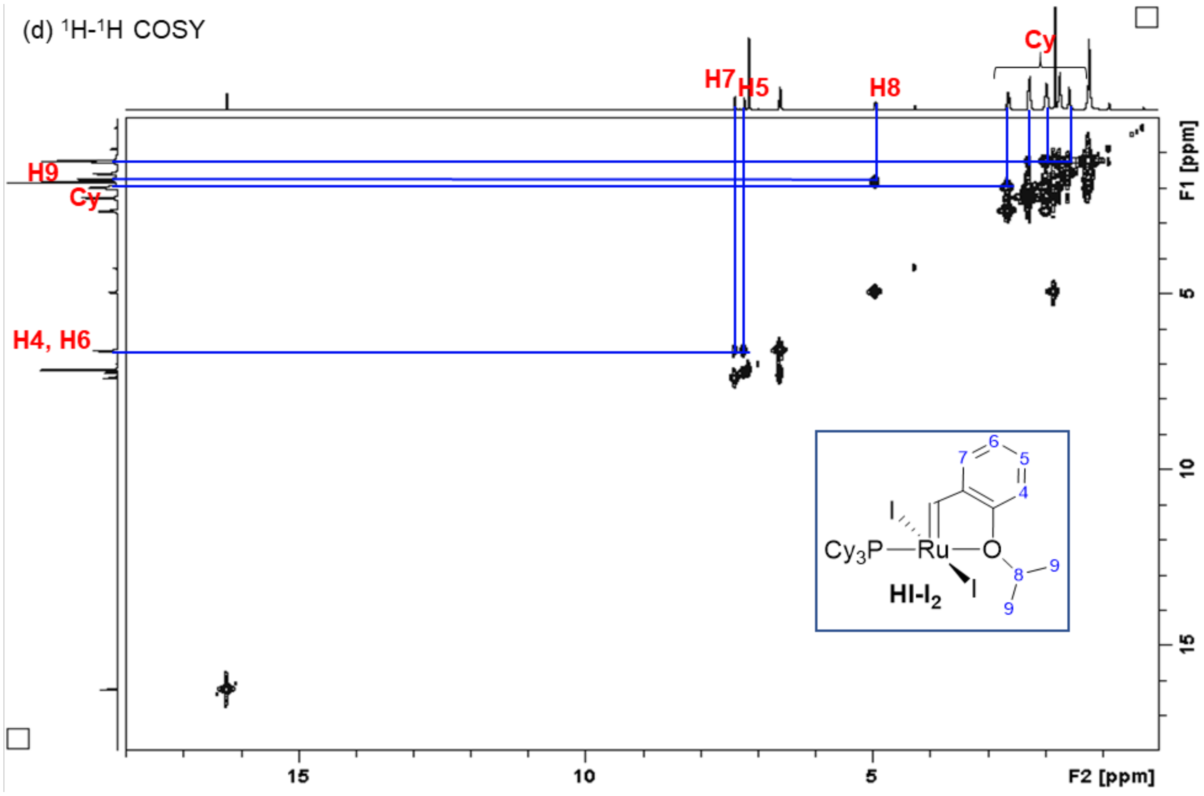

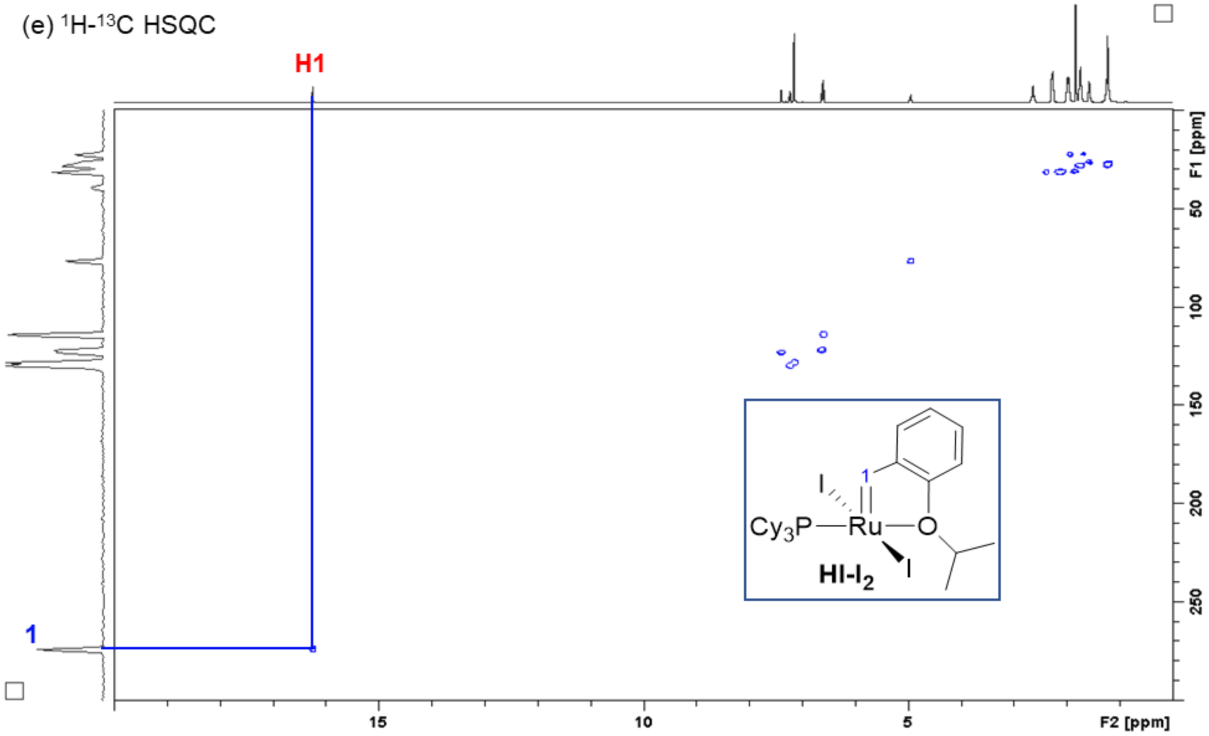

Figure S1. NMR spectra (C_6D_6) for synthetic intermediate **HI-I$_2$**. (a) ^{1}H NMR (500 MHz; inset shows alkylidene signal. (b) ^{13}C{^{1}H} NMR (125 MHz; alkylidene C not observed; located by ^{1}H-^{13}C HSQC. (c) ^{31}P{^{1}H} NMR (200 MHz). (d) ^{1}H-^{1}H COSY NMR (500 MHz). (e) ^{1}H-^{13}C HSQC NMR spectrum (500 MHz, 125 MHz; spectrum on vertical axis is projection of cross-peak).

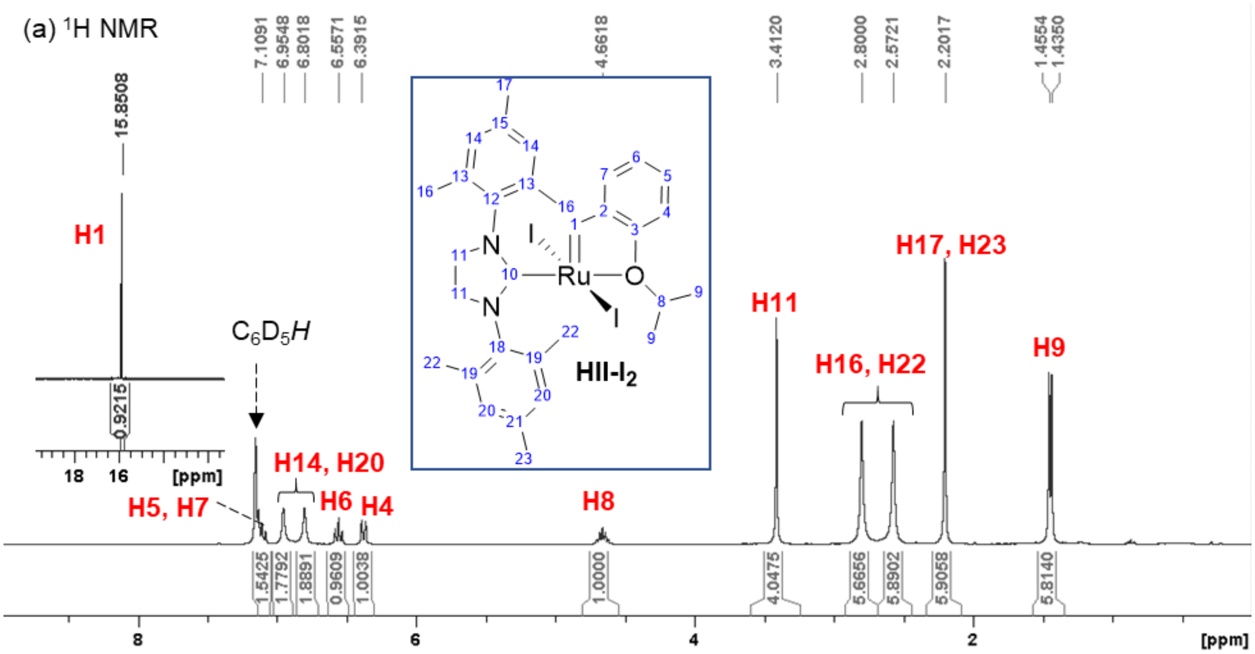

Figure S2. ^{1}H NMR spectrum (500 MHz, C_6D_6) of known **HII-I$_2$**, prepared from **HI-I$_2$** by ligand exchange with H_2IMes. Inset shows the signal for the alkylidene [Ru]=C*H*Ar proton.

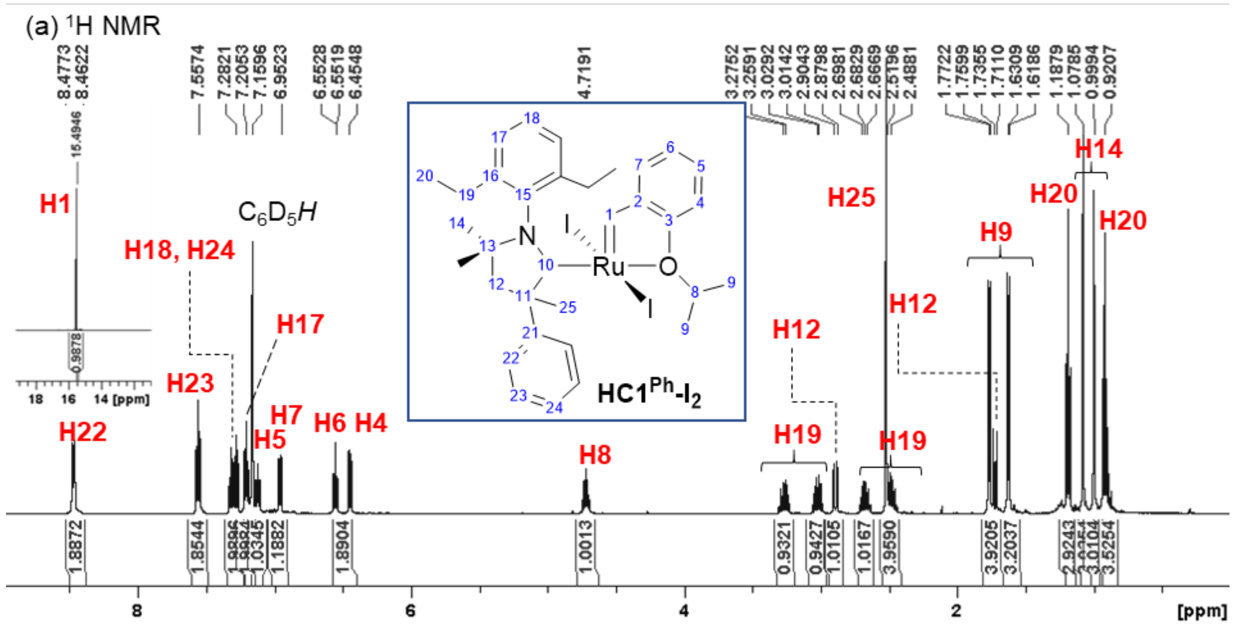

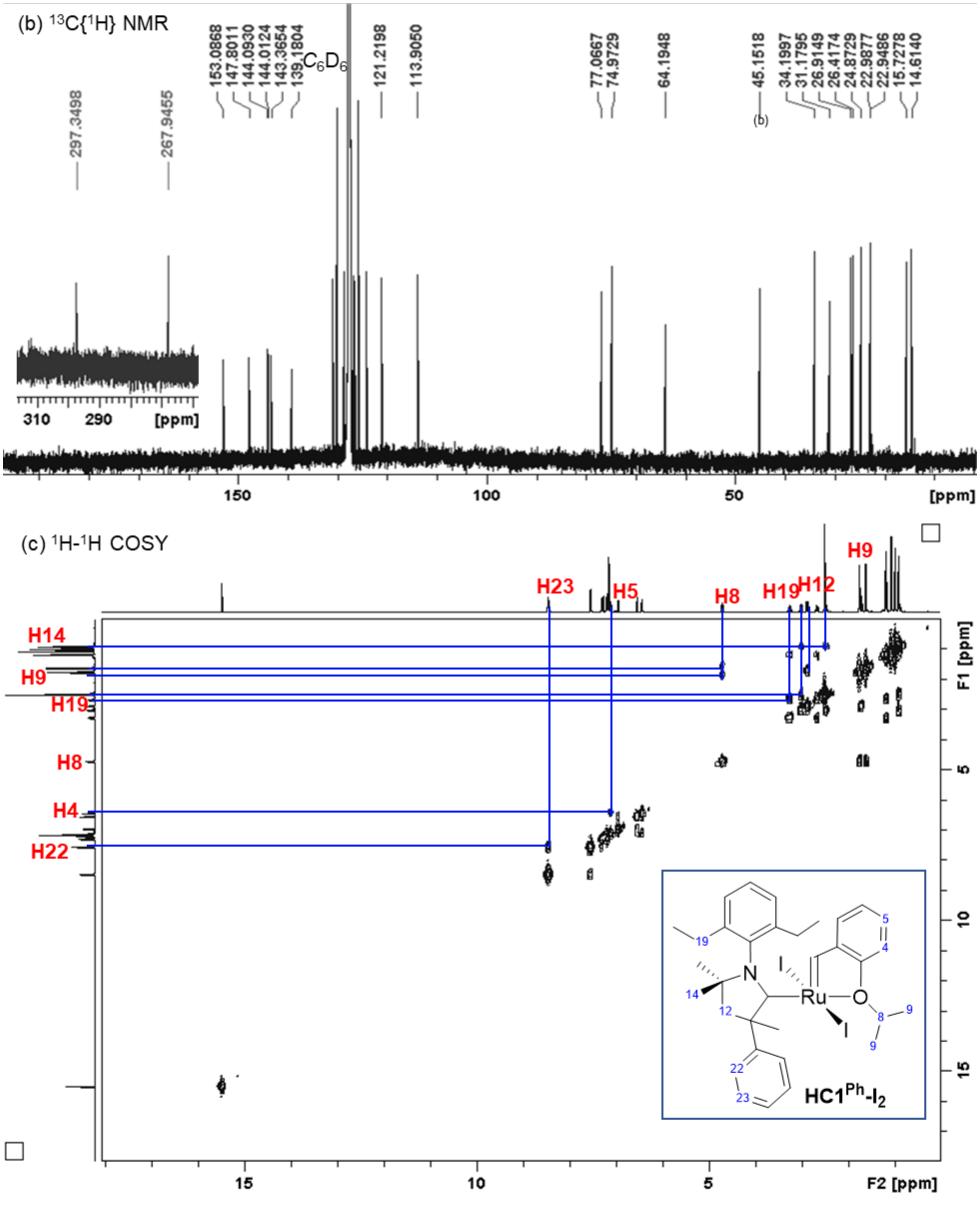

Figure S3. NMR spectra (C_6D_6) for **HC1^{Ph}-I$_2$**. (a) ^{1}H NMR (500 MHz; inset shows alkylidene signal. (b) ^{13}C{^{1}H} NMR (125 MHz). (c) ^{1}H-^{1}H COSY NMR (500 MHz).

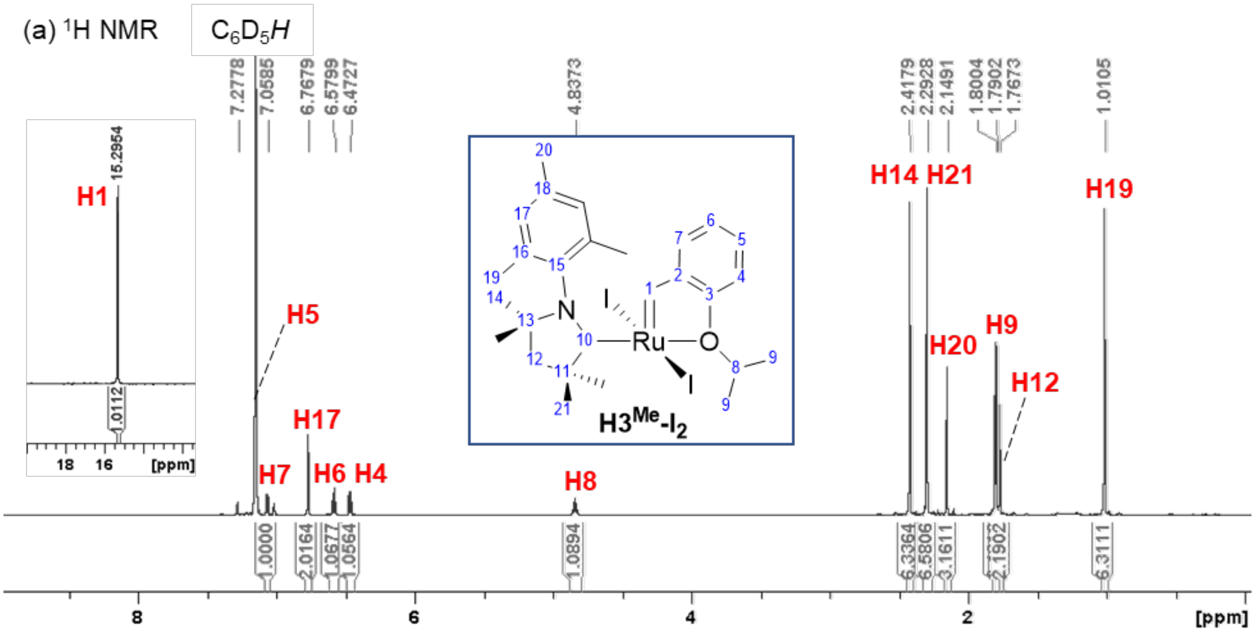
(a) ¹H NMR
C6D5H
15.2954
H1
1.0112
18 16 [ppm]
7.2778
7.0585
6.7679
6.5799
6.4727
4.8373
2.4179
2.2928
2.1491
1.8004
1.7902
1.7673
1.0105
H5
H17
H7
H6 H4
H8
H14 H21
H20
H9
H12
H19
H3Me-I2
1.0000
2.0164
1.0677
1.0564
1.0894
6.3364
6.5806
3.1611
2.1902
6.3111
8
6
4
2
[ppm]

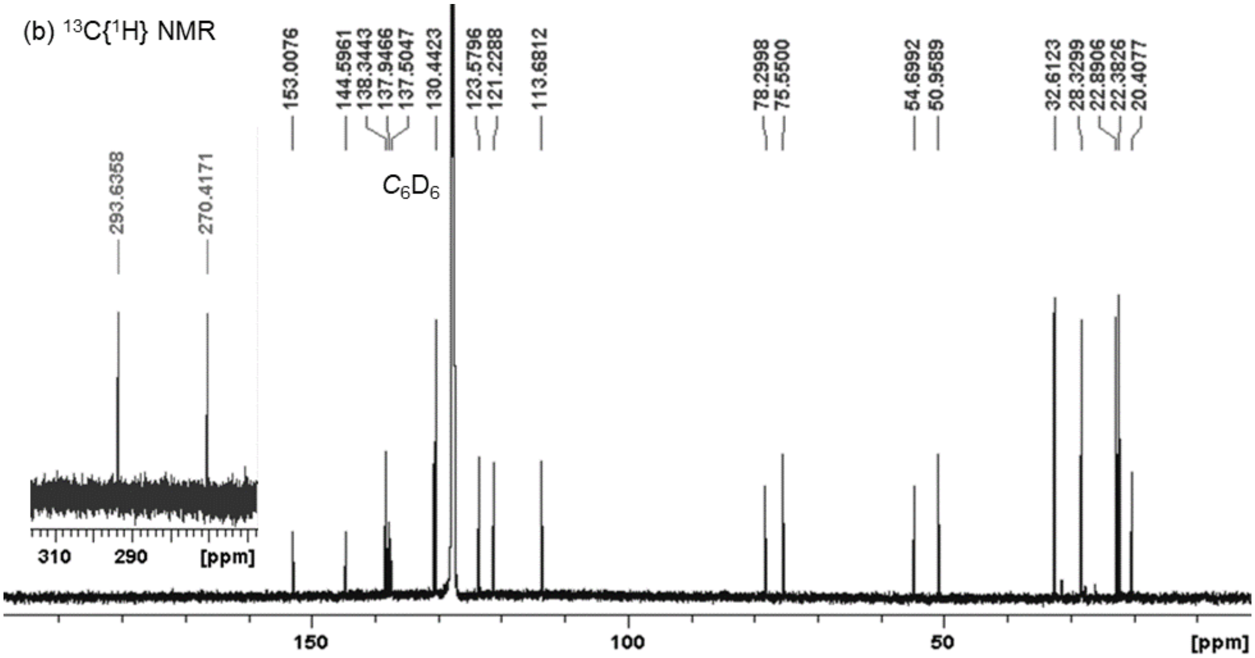
(b) ¹³C{¹H} NMR
293.6358
270.4171
310 290 [ppm]
153.0076
144.5961
138.3443
137.9466
137.5047
130.4423
123.5796
121.2288
113.6812
78.2998
75.5500
54.6992
50.9589
32.6123
28.3299
22.8906
22.3826
20.4077
C6D6
150
100
50
[ppm]

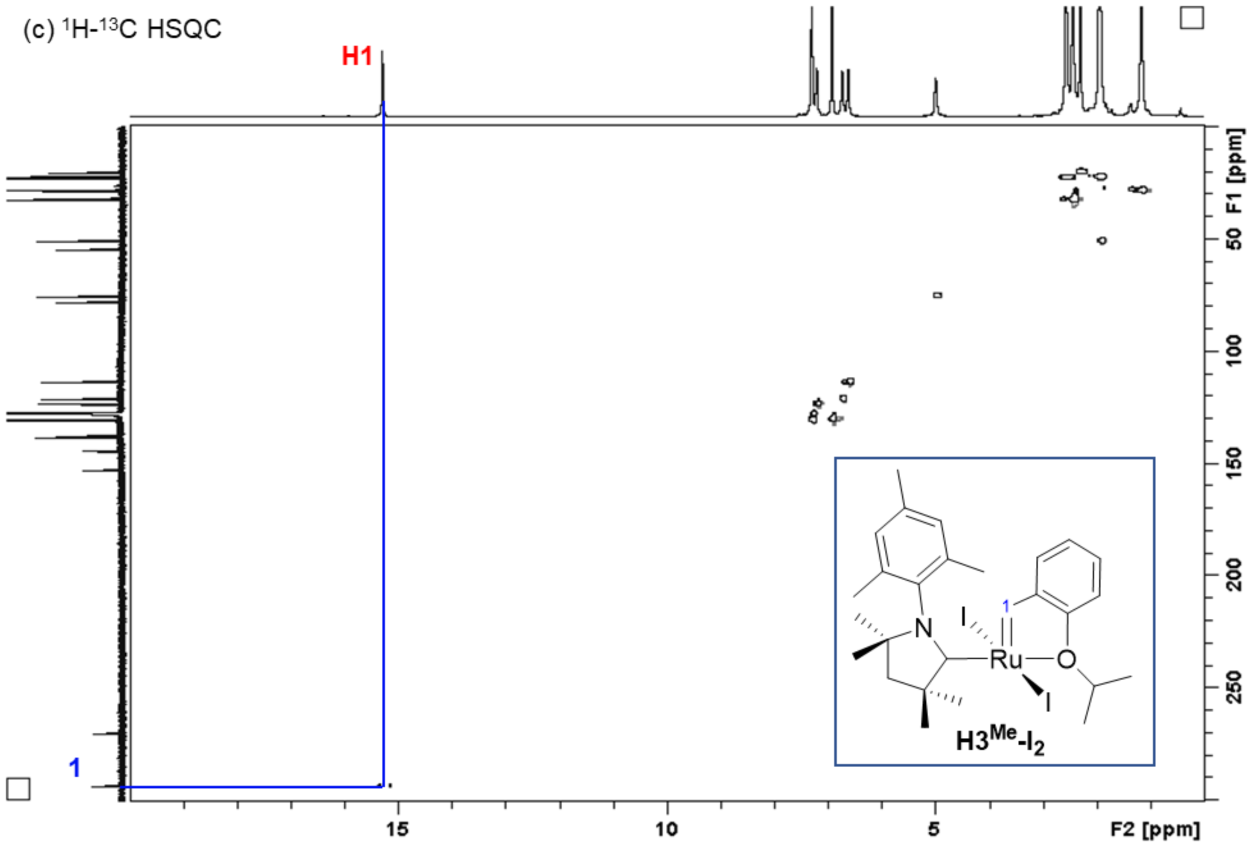

Figure S4. NMR spectra (C_6D_6) for **HC3^{Me}-I$_2$**. (a) ^{1}H NMR (600 MHz; inset shows alkylidene signal). Spinning side-bands flank the C_6D_5*H* singlet. (b) ^{13}C{^{1}H} NMR (125 MHz). (c) ^{1}H-^{13}C HSQC NMR (500 MHz, 125 MHz).

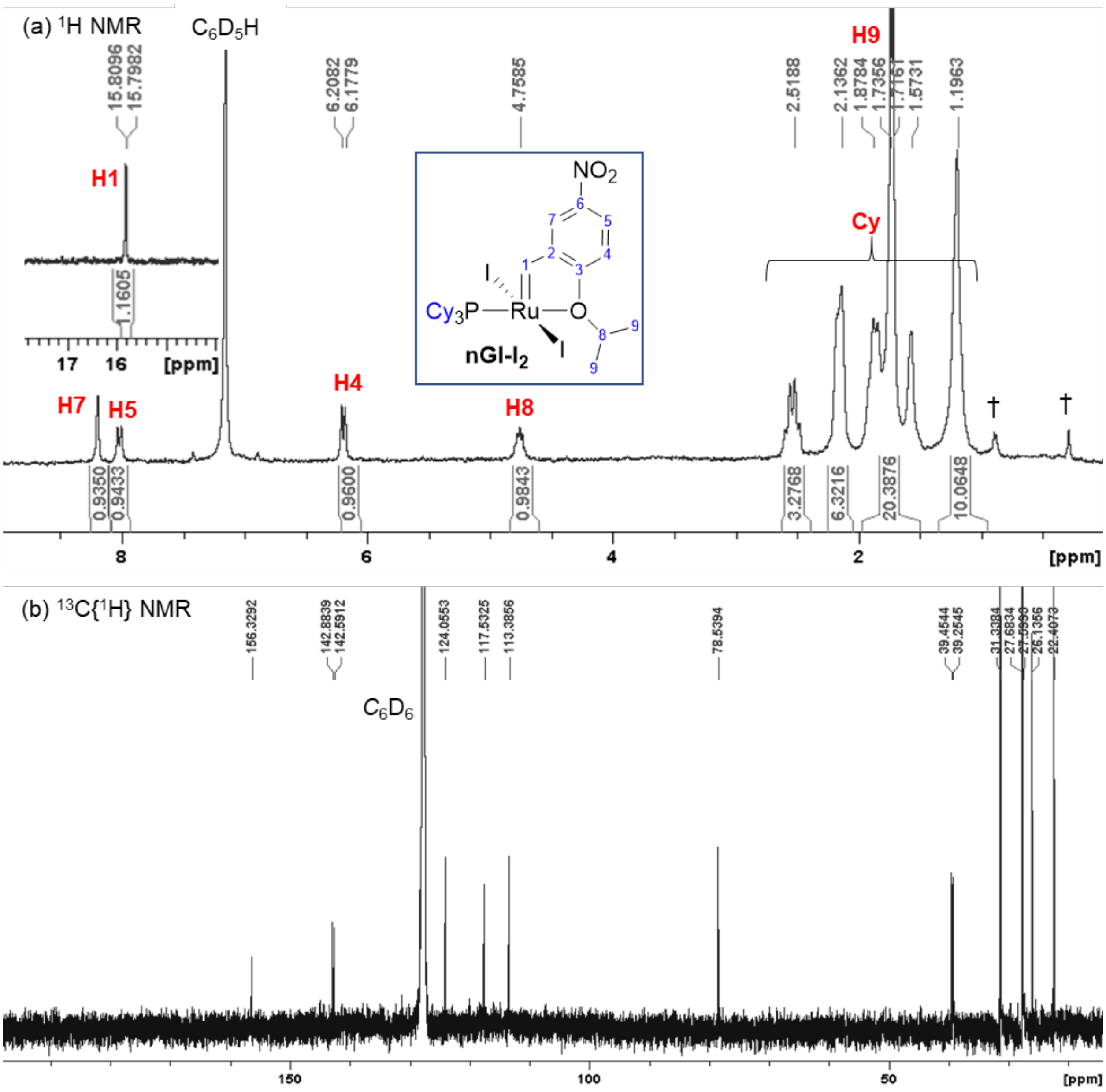
(a) 1H NMR
C6D5H
H9
15.8096
15.7982
6.2082
6.1779
4.7585
2.5188
2.1362
1.8784
1.7356
1.5731
1.1963
H1
NO2
Cy
Cy3P
Ru
O
I
nGI-I2
1.1605
17
16
[ppm]
H7
H5
H4
H8
†
†
0.9350
0.9433
0.9600
0.9843
3.2768
6.3216
20.3876
10.0648
8
6
4
2
[ppm]
(b) 13C{1H} NMR
156.3292
142.8839
142.5912
124.0553
117.5325
113.3856
78.5394
39.4544
39.2545
31.3384
27.6834
26.1356
C6D6
150
100
50
[ppm]

(c) $^{31}P\{^{1}H\}$ NMR

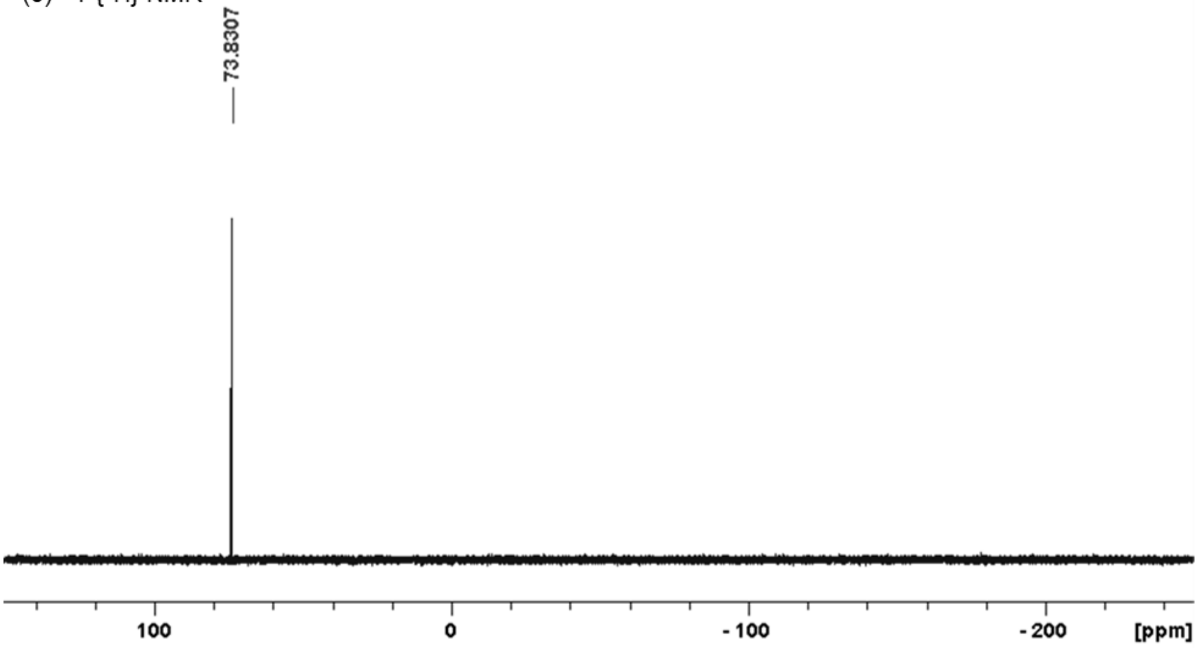

(d) ^{1}H-^{1}H COSY

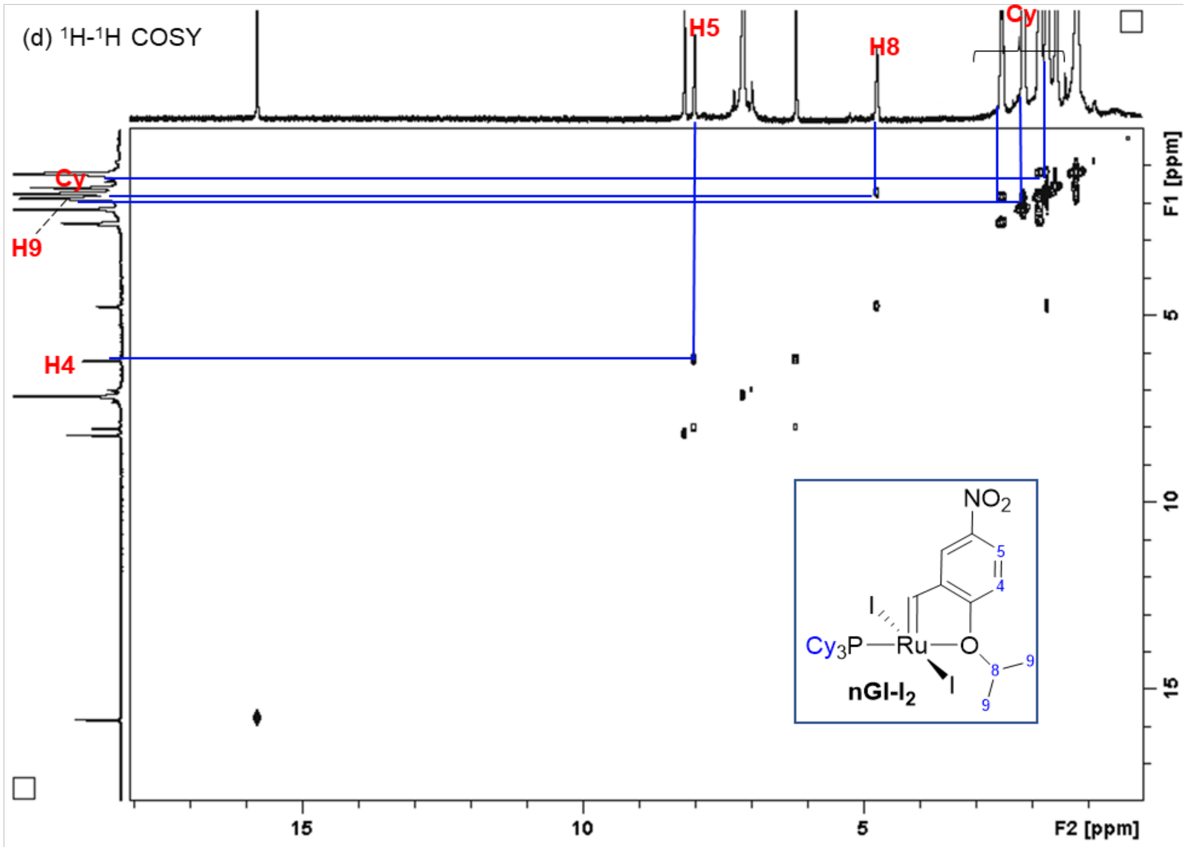

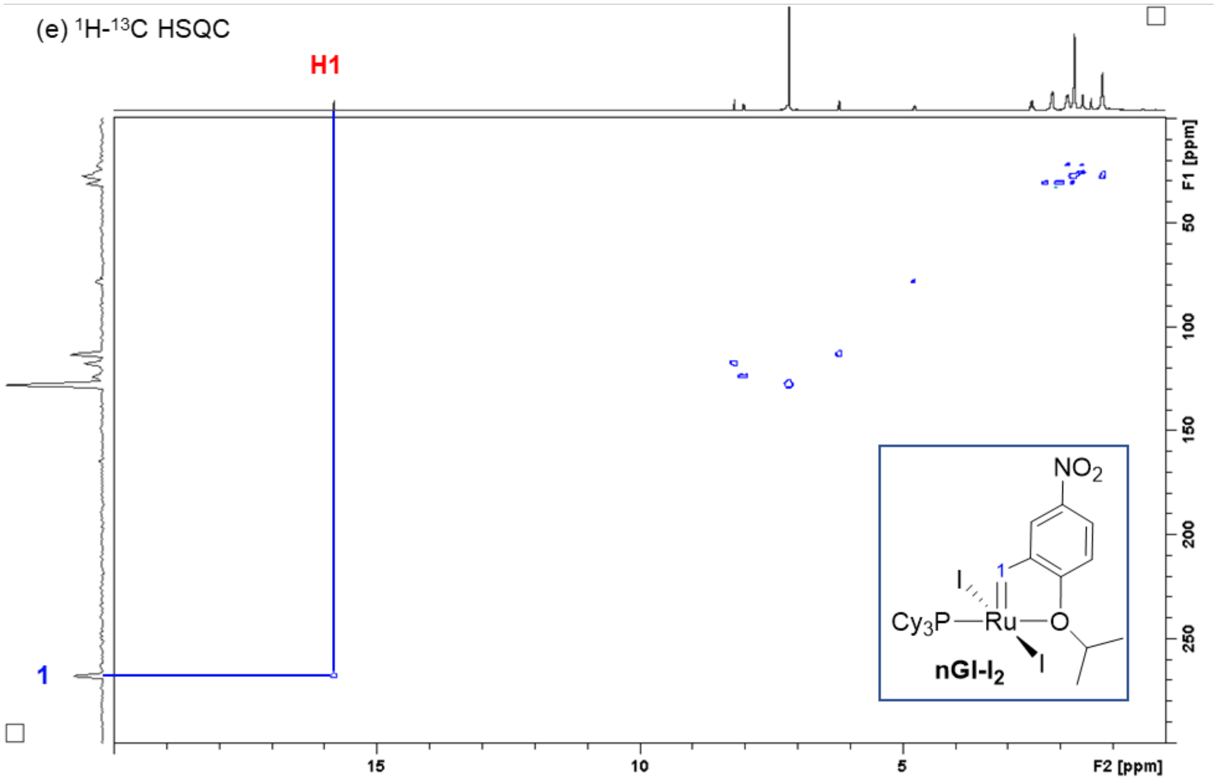

Figure S5. NMR spectra (C_6D_6) for synthetic intermediate **nGI-I$_2$**. (a) ^{1}H NMR (300 MHz; inset shows alkylidene signal). Trace silicone grease and THF indicated by (†). (b) ^{13}C{^{1}H} NMR (125 MHz). Alkylidene carbon not observed; located by ^{1}H-^{13}C HSQC. (c) ^{31}P{^{1}H} NMR (200 MHz). (d) ^{1}H-^{1}H COSY NMR (500 MHz). (e) ^{1}H-^{13}C HSQC NMR (500 MHz, 125 MHz).

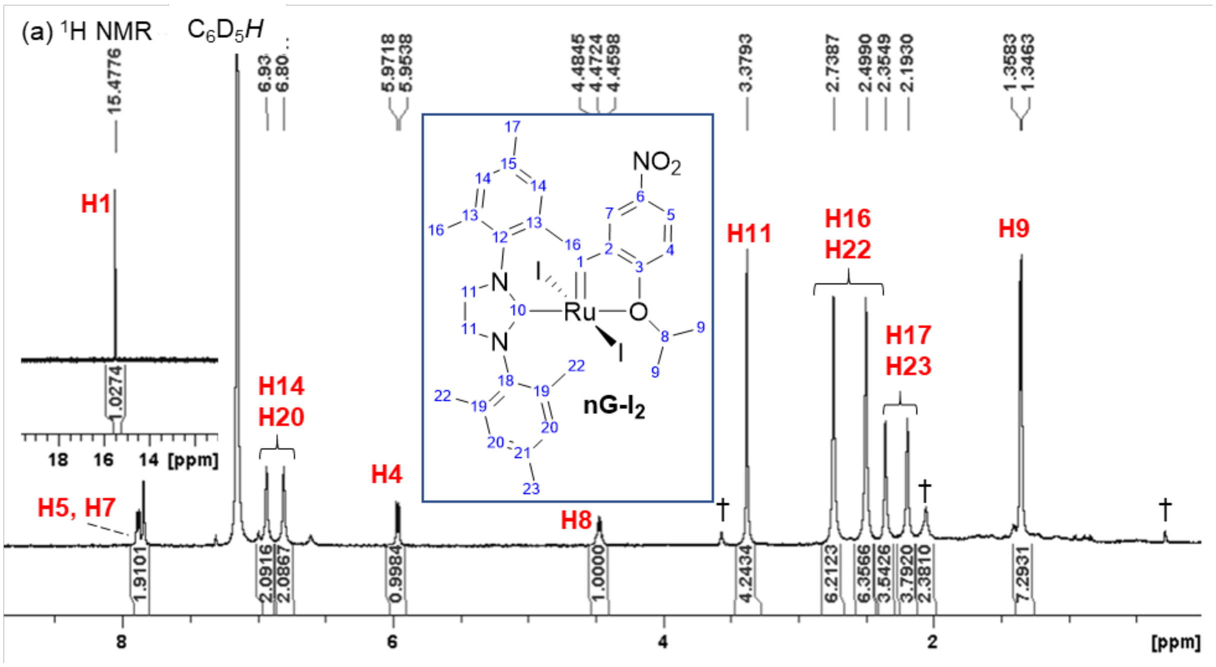

Figure S6. ^{1}H NMR spectrum (500 MHz, C_6D_6) of known **nG-I$_2$**. Inset shows the signal for the alkylidene [Ru]=C*H*Ar proton. Trace grease and solvent indicated by (†).

S3. References.

(1) Schwab, P.; Grubbs, R. H.; Ziller, J. W., Synthesis and Applications of $RuCl_2(=CHR')(PR_3)_2$: The Influence of the Alkylidene Moiety on Metathesis Activity. *J. Am. Chem. Soc.* **1996**, *118*, 100–110.

(2) Gawin, R.; Kozakiewicz, A.; Guńka, P. A.; Dąbrowski, P.; Skowerski, K., Bis(Cyclic Alkyl Amino Carbene) Ruthenium Complexes. *Angew. Chem., Int. Ed.* **2017**, *56*, 981–986.

(3) Van Veldhuizen, J. J.; Gillingham, D. G.; Garber, S. B.; Kataoka, O.; Hoveyda, A. H., Chiral Ru-based complexes for asymmetric olefin metathesis: Enhancement of catalyst activity through steric and electronic modifications. *J. Am. Chem. Soc.* **2003**, *125*, 12502–12508.

(4) Fürstner, A.; Langemann, K., Macrocycles by Ring-Closing Metathesis. *Synthesis* **1997**, 792–803.

(5) Nascimento, D. L.; Davy, E. C.; Fogg, D. E., Merrifield Resin-Assisted Routes to Second-Generation Catalysts for Olefin Metathesis. *Catal. Sci. Technol.* **2018**, 1535–1544.

(6) Arduengo, A. J.; Krafczyk, R.; Schmutzler, R.; Craig, H. A.; Goerlich, J. R.; Marshall, W. J.; Unverzagt, M., Imidazolylidenes, imidazolinylidenes and imidazolidines. *Tetrahedron* **1999**, *55*, 14523–14534.

(7) Wappel, J.; Urbina-Blanco, C. A.; Abbas, M.; Albering, J. H.; Saf, R.; Nolan, S. P.; Slugovc, C., Halide Exchanged Hoveyda-Type Complexes in Olefin Metathesis. *Beilstein J. Org. Chem.* **2010**, *6*, 1091–1098.

(8) Tracz, A.; Matczak, M.; Katarzyna; Urbaniak; Skowerski, K., Nitro-Grela-Type Complexes Containing Iodides – Robust and Selective Catalysts for Olefin Metathesis Under Challenging Conditions. *Beilstein J. Org. Chem.* **2015**, *11*, 1823–1832.
